# Per capita sugar consumption and prevalence of diabetes mellitus – global and regional associations

**DOI:** 10.1186/1471-2458-14-186

**Published:** 2014-02-20

**Authors:** Praveen Weeratunga, Sayumi Jayasinghe, Yashasvi Perera, Ganga Jayasena, Saroj Jayasinghe

**Affiliations:** 1 University Medical Unit, National Hospital of Sri Lanka, Colombo, Sri Lanka; 2 Western Health Australia, Footscray, Australia; 3 Ministry of Health, Colombo, Sri Lanka; 4 Department of Clinical Medicine, University of Colombo, Colombo, Sri Lanka

## Abstract

**Background:**

Diabetes mellitus (DM) is a rampant epidemic worldwide. Causative factors and predisposition is postulated to be multi-factorial in origin and include changing life styles and diet. This paper examines the relationship between per capita sugar consumption and diabetes prevalence worldwide and with regard to territorial, economic and geographical regions.

**Methods:**

Data from 165 countries were extracted for analysis. Associations between the population prevalence of diabetes mellitus and per capita sugar consumption (PCSC) were examined using Pearson’s correlation coefficient (PCC) and multivariate linear regression analysis with, infant mortality rates (IMR, as an general index maternal and child care), low birth weight (LBW, as an index of biological programming) and obesity prevalence included in the model as confounders.

**Results:**

Despite the estimates for PCSC being relatively crude, a strong positive correlation coefficient (0.599 with p < 0.001) was observed between prevalence of diabetes mellitus and per capita sugar consumption using data from all 165 countries. Asia had the highest correlation coefficient with a PCC of 0.660 (p < 0.001) with strongest correlation noted in Central (PCC = 0.968; p < 0.001), South (PCC = 0.684; p = 0.050) and South East Asia (PCC = 0.916; p < 0.001). Per capita sugar consumption (p < 0.001; Beta = 0.360) remained significant at the last stage as associations of DM prevalence (R^2^ = 0.458) in the multivariate backward linear regression model. The linear regression model was repeated with the data grouped according to the continent. Sugar was noted to be an independent association with DM only with regard to Asia (p < 0.001 Beta = 0.707) and South America (p = 0.010 Beta 0.550). When countries were categorized based on income PCS and DM demonstrated significant association only for upper middle income countries (p < 0.001 Beta 0.656).

**Conclusions:**

These results indicate independent associations between DM prevalence rates and per capita sugar consumption both worldwide and with special regard to the Asian region. Prospective cohort studies are proposed to explore these associations further.

## Background

The world is facing an unprecedented epidemic of type 2 diabetes mellitus (DM). Global estimated prevalence for the disease is at 6.4% (285 million adults) in 2010 and 7.7% (439 million adults) for 2030. The estimated rise in prevalence is more marked among developing countries [1].

The causes for this epidemic are multi-factorial and include an ageing population, genetic and environmental factors. Of the latter, changing life styles (diet, sedentary life styles), low birth weight (i.e. biological programming), obesity, and poverty are considered important.

Of the life style factors, prevalence is related to excessive weight gain in infancy and subsequent obesity and thereby obesity [2]. Age and living in an urban environment are major determinants of diabetes among South Kivu Congolese adults [3] and in Sri Lanka [4].

Studies from Taiwan have reported higher incidence of diabetes among poorer groups [5]. However, similar results are not seen in other areas such as southern Asia (for example in India and in Sri Lanka), where prevalence of diabetes is higher in the more affluent groups [4,6,7].

Diet related factors contributing to diabetes include carbohydrate load and vitamin D status [8].

A recent meta-analysis of prospective cohort studies have shown a significant positive associations between dietary glycaemic index and glycaemic load and the risk of type 2 diabetes [9,10]. Consumption of sugar sweetened beverages has been shown to predispose to the development of type 2 DM in several large observational studies from the US [11]. Similar associations have been noted in the recently published study based on a French cohort [12].

A few studies have investigated the influence of sugar intake at international level.

Population prevalence of diabetes from 173 countries was found to be positively related to sugar exposure [13]. Duration and degree of sugar exposure was found to correlate with diabetes prevalence in a dose-dependent manner, while declines in sugar exposure correlated with significant subsequent declines in diabetes rates independently of other socioeconomic, dietary and obesity prevalence changes [14].

There are significant variations in the prevalence of diabetes among regions. For example, the southern Asian and Pacific regions are well known to have a high rate of diabetes. Therefore we hypothesized that the impact of sugar consumption rates on prevalence of DM may vary among the different regions of the world. This paper examines this hypothesis using prevalence rates of DM and per capita sugar consumption. The possible explanatory model took the following to account based on Barker’s hypothesis: infants who had a lower birth weight mainly due to maternal deprivation (assessed by low birth weight and poverty rates respectively) and then chronically undernourished during infancy and early childhood (reflected with infant mortality rate), begin to take high calorie food after weaning and as young adults (where per-capita sugar consumption is a proxy measure) then become obese and diabetic.

Furthermore the conceptual basis to the hypothesis that refined sugar promotes DM by leading to obesity and insulin resistance and the other possible mechanism is that refined sugar, due to rapid intestinal absorption induces hyper-secretion of insulin from beta cells that subsequently lead to their exhaustion and sub-optimal secretion.

Therefore this study explores the associations of per capita consumption of sugar with the worldwide prevalence of DM. It furthermore aims to exhibit special emphasis on regional variations based on continent, region, economic and trade related factors and income strata. This attempts to capture inter regional differences in socio economic status and to a lesser extent racial differences.

## Methods

Data from 165 countries were selected for analysis. Country selection was based on data availability for all variables selected for study. Countries where data was incomplete were excluded. The prevalence rates for diabetes were obtained from the database of the International Diabetes Federation (IDF) for the year 2011 [15]. Per-capita sugar intake was obtained for the year 2005 from the Sugar Year Book 2010 [16].

The per capita sugar intake is defined as the raw sugar consumption per person of a given country or territory. This is calculated based on the statistical disappearance of sugar in the country or territory after adjustment for trade and exports. The assumption is made that the statistical disappearance of sugar is equal to consumption after adjusting for utilization for non human consumption.

The selection of data for analysis was based on fulfillment of the criteria stated below;

a) Completeness of data across all analyzed variables

b) The most updated and recent datasets available.

Hence DM prevalence data was selected from 2011. Sugar consumption was backdated to reflect exposure with delayed presentation.

The categorization of countries was done based on (a) regional classification of the World Health Organization [17] (b) economic regions, (c) continents and (d) income strata. Continents were classified based on the United Nations macro geographical (continental) regions, geographical sub-regions, and selected economic and other groupings [18]. Economic regions and income strata based on gross domestic product (GDP) were classified based on the world economic outlook databases of the International Monetary Fund [19]. All data was extracted from freely available databases.

Analysis was done using the population prevalence of diabetes mellitus as the outcome variable, and the following as explanatory variables as explained in the previous section: per capita sugar consumption, infant mortality rates (IMR, as a general index maternal and child care), low birth weight (LBW, as an index of biological programming), obesity and over-weight rates, and gross national income (to indicate affluence) and poverty rates.

IMR, LBW data backdated by 20 years (1985) was used to reflect exposure to current DM prevalence. Obesity and overweight rates were obtained from the WHO databases [20,21].

Pearson’s correlation coefficients (PCC) were calculated for the outcome variables and multivariate linear regression analysis was done to describe the relationships between the outcome variable and the explanatory variables. Further sub analysis was performed based on geographic, economic and regional classifications.

## Results

Data was analyzed for a total of 165 countries. (n = 165), excluding Botswana which was an extreme outlier.

A strong positive correlation coefficient (0.599; p < 0.001) was observed between prevalence of diabetes mellitus and per capita sugar consumption using data from all 165 countries.

Table 1 presents correlation data based on continents and subcontinents based on the United Nations (UN) territorial classification. Asia had the highest correlation coefficient with a PCC of 0.660 (p < 0.001) and lower correlations were observed for Africa. Within Asia strongest correlation was seen in Central, South and South-East Asian countries. The Eastern European region demonstrated a positive correlation between PCSC and DM prevalence whereas other European regions did not.

**Table 1:** Correlation coefficients of per capita sugar consumption and DM based on continents and regions

**Correlation**		**Pearson’s correlation coefficient**	**Significance**
General		0.599	< 0.001
Continents and Regions			
Africa		0.381	0.007
North America		0.285	0.238
South America		0.410	0.186
Australia/Oceania		0.796	0.107
Asia		0.660	< 0.001
	South Asia	0.684	0.050
	South East Asia	0.916	< 0.001
	Central Asia	0.968	< 0.001
	Eastern Asia	0.792	0.060
	Western Asia	0.088	0.822
Europe		0.287	0.069
	Northern	-0.165	0.649
	Eastern	0.608	0.036
	Southern	0.384	0.218
	Western	0.097	0.836

These correlations are also reflected in analysis based on the WHO regions with South East Asia (SEARO) (PCC = 0.786 p = 0.012) and Western Pacific (WPRO) (PCC = 0.722 p =0.001) regions showing increased correlation of per capita sugar consumption with DM (Table 2).

**Table 2:** Correlation coefficients between Per capita sugar consumption and Diabetes prevalence based on WHO region

	**Pearson’s correlation coefficient**	**Significance**
AFRO	0.273	0.080
EURO	0.433	0.060
AMRO	0.372	0.039
EMRO	0.478	0.052
SEARO	0.786	0.012
WPRO	0.722	0.001

Table 3 presents the correlations based on economy status as defined by the GDP. Low middle income countries exhibited significant positive correlation with diabetes prevalence. (PCC = 0.468, p =0.002).

**Table 3:** Correlation coefficients between per capita sugar consumption and DM – based on the income classification

**Income status**	**Pearson’s correlation coefficient**	**Significance**
Low	0.168	0.335
Low middle	0.468	0.002
Upper middle	0.108	0.490
High	0.123	0.467

A backward linear regression model was applied to investigate further the relationship of per capita sugar consumption to worldwide prevalence of DM (Table 4). Curve estimation of the relationship between per capita sugar consumption and diabetes prevalence demonstrated linearity. Infant mortality rates (IMR, as a general index maternal and child care), low birth weight (LBW, as an index of biological programming), obesity and over-weight rates, and gross national income (to indicate affluence) and poverty rate were used as independent variables in the model.

**Table 4:** Independent predictors of DM prevalence based on multivariate linear regression modeling

	**Variable**	**Beta**	**Significance**	**Model R**^**2**^
N = 165	PCSC	0.360	0.001	0.458
	Obesity	0.256	0.004	
	Low birth weight	0.216	0.005	
	IMR	-0.383	0.001	

Per capita sugar consumption (p < 0.001; Beta = 0.360) remained significant at the last stage as associations of DM prevalence (R^2^ = 0.458). The linear regression model was repeated with the data grouped according to the continent. Sugar was noted to be an independent association with DM only with regard to Asia (p < 0.001 Beta = 0.707) and South America (p = 0.010 Beta 0.550) R^2^ = 0.568.

When countries were categorized based on income PCS and DM demonstrated significant association only for upper middle income countries (p < 0.001 Beta 0.656) R^2^ = 0.467. In this sub analysis obesity was noted as a significant association of DM in low and middle income countries. (p = 0.047 Beta 0.367) and p = 0.019 Beta 0.371) R^2^ = 0.545.

Per capita sugar consumption showed a positive correlation with economy (PCC = 0.604; p < 0.001) and male (PCC = 0.580; p < 0.001) and female obesity (PCC = 0.579; p < 0.001).

## Discussion

**Figure 1:**
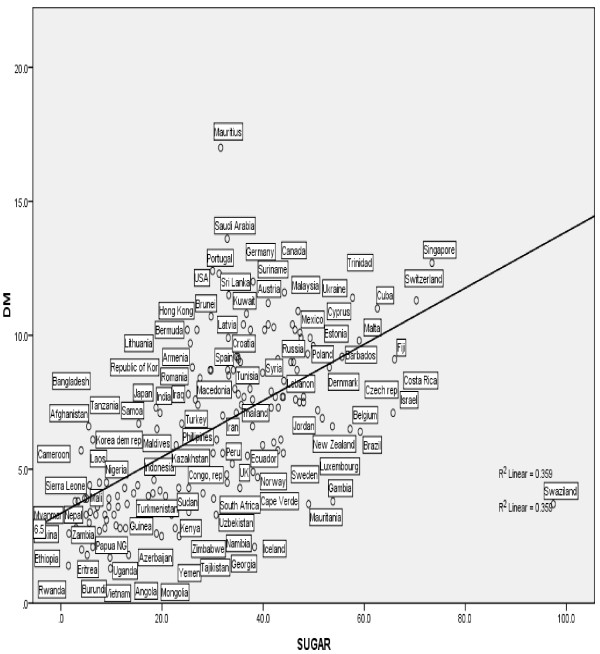
**Linear regression plot of PCSC and diabetes mellitus (R**^**2**^ **= 0.458; p =0.001).**

Our results indicate that at the country-level, gross per capita consumption of sugar correlates with diabetes prevalence. Furthermore, per-capita sugar consumption is an independent predictor of diabetes prevalence after adjusting for potential confounders including obesity and overweight (p < 0.001; Beta = 0.360) (R^2^ = 0.458). The relationship was also noted to be linear with a strong correlation (Figure 1).

Furthermore the results indicate that the correlation of sugar consumption to DM varies based on socio – economic status (Table 3) and geographical location (Table 1). A prominent correlation was noted between diabetes and sugar consumption in the Asia Pacific region [0.660 (p < 0.001)]. It is also notable that in this region the contribution of obesity rates towards DM prevalence seems minimal as reflected by the low correlation coefficients.

There is special interest in this region as it has many unique features in diabetes prevalence. These include a rapid increase in DM prevalence in the region, especially pronounced in the South Asian region. This has been attributed to genetic predisposition, rapid urbanization and socio-economic transition. It is also noteworthy that patients in the region develop DM at a younger age and at a lower threshold of BMI [22]. This has led research to identify other non conventional risk factors to account for DM in this region. It is possible based on the results of the above study that sugar consumption which may play a significant role towards DM prevalence in the region independent of obesity. This is also reflected in the linear regression model where in the Asian region sugar was retained an independent association of DM.

The strong association of sugar consumption and DM prevalence may reflect the rapid urbanization in the region. Other contextual factors such as alteration of lifestyle, dietary practices, and environmental factors may play a role in the above association.

It is important to examine possible postulations for the observations discussed above which highlight the independent association between PCSC and DM prevalence. It is possible that pathways independent of obesity are playing a role in the possible pathogenesis of these associations. Sugar has been hypothesized to predispose towards DM by several mechanisms. These include obesity dependent mechanisms [23] as well as alternate pathways such as the impact of fructose on unregulated hepatic lipogenesis and fatty acid oxidation which subsequently leads to inactivation of the insulin signaling pathway [24]. Sugar has also been shown to induce insulin resistance [25] and pancreatic beta cell destruction due to reactive oxygen species produced during metabolism [26]. It is possible that in regions such as South Asia with a strong correlation between sugar consumption and DM that genetic influence in residents may create predilection for prominence of these metabolic pathways.

Similar associations between sugar consumption and diabetes have been observed by Basu et al. [13] determining that total joules attributed to sugar and sweeteners was associated with diabetes prevalence based on data from 173 countries. Furthermore duration and degree of sugar exposure was found to be associated in a dose dependent manner independent of other confounders such as in another similar study [14]. These studies have utilized food supply data which expresses the amount of food available for human consumption over a reference period and extracted total joules related to sugar and other sweeteners as an index of sugar consumption. Per capita supplies represent only the average supply available for each individual in the population as a whole and do not indicate what is actually consumed by individuals.

In contrast, the index of sugar consumption in our analysis was extracted from the raw data of total per capita sugar consumption. This reflects the total statistical disappearance of sugar within the country concerned with the assumption that this sugar was consumed by the individuals of the particular country. However this also includes sugar for manufacture of sugar containing products for export and other sugar containing food other than for human consumption (this variable was excluded wherever data was available).

PCSC was found to be independently associated with DM prevalence in upper middle income countries. This was not the case in lower middle and low income countries where obesity was noted to be the independent association of DM prevalence. This indicates that in upper middle income countries sugar consumption may have an impact on DM prevalence independent of obesity.

Our study has a few limitations. The prevalence values for DM were extracted from the IDF database. These values include patients with Type 1 DM which may impact the final results. It is probable that datasets from developing countries are less complete than those from developed countries due to issues of under diagnosis. Furthermore some data, especially those for African countries and former soviet states are estimated and may differ from the true value.

Per capita sugar consumption reflects a raw marketplace value and data available for some countries are only estimates. Furthermore other confounders were not taken into account in this study may play a role in DM prevalence (e.g. stress).

Within the constraints of the study we cannot conclude causality as this is only cross sectional data. Other unestimated confounders and contextual factors may be at play.

Longitudinal examination of variations in sugar consumption and DM prevalence adjusting for changes in other factors such as poverty, affluence and urbanization could provide better insight into causality but we encountered several limitations due to inadequate data.

This study creates avenues for further study into the subject with exposure based longitudinal cohort studies. This will in turn create far reaching public health implications in DM and its prevention. The findings of this study and previous similar studies could be a basis for the implementation of removal of subsidies to sugar cane plantations, increased taxes on sugar and foods that use sugar, and limits on the sugar contents in drinks and other beverages to battle the world wide epidemic of DM.

## Conclusions

Per capita sugar consumption is independently associated with DM prevalence worldwide with a predilection noted in the Asian region. The mechanisms for which should be further elucidated. Direct causality cannot be determined and prospective cohort studies are recommended.

## Competing interests

All authors (PW, SayJ, YP, GJ, and SJ) declare to have no financial or non financial competing interests.

## Authors’ contributions

SJ, SayJ, YP were involved in conception of the project. PW and GJ were involved in preparation of methodology and analysis of data. PW and SJ were involved in preparation of the manuscript. All authors read and approved the final manuscript.

## Pre-publication history

The pre-publication history for this paper can be accessed here:

http://www.biomedcentral.com/1471-2458/14/186/prepub
